# Differential gene expression patterns in cyclooxygenase-1 and cyclooxygenase-2 deficient mouse brain

**DOI:** 10.1186/gb-2007-8-1-r14

**Published:** 2007-01-31

**Authors:** Christopher D Toscano, Vinaykumar V Prabhu, Robert Langenbach, Kevin G Becker, Francesca Bosetti

**Affiliations:** 1 Brain Physiology and Metabolism Section, National Institute on Aging, National Institutes of Health, Bldg. 9, Rm. 1S126, 9 Memorial Drive, Bethesda, Maryland 20892, USA; 2 Gene Expression and Genomics Unit, National Institute on Aging, National Institutes of Health, Gerontology Research Center, 5600 Nathan Shock Drive, Baltimore, Maryland, 21224, USA; 3 Laboratory of Molecular Carcinogenesis, National Institute of Environmental Health Sciences, National Institutes of Health, 111 TW Alexander Drive, Research Triangle Park, North Carolina, 27709, USA

## Abstract

**Background:**

Cyclooxygenase (COX)-1 and COX-2 produce prostanoids from arachidonic acid and are thought to have important yet distinct roles in normal brain function. Deletion of COX-1 or COX-2 results in profound differences both in brain levels of prostaglandin E_2 _and in activation of the transcription factor nuclear factor-κB, suggesting that COX-1 and COX-2 play distinct roles in brain arachidonic acid metabolism and regulation of gene expression. To further elucidate the role of COX isoforms in the regulation of the brain transcriptome, microarray analysis of gene expression in the cerebral cortex and hippocampus of mice deficient in COX-1 (COX-1^-/-^) or COX-2 (COX-2^-/-^) was performed.

**Results:**

A majority (>93%) of the differentially expressed genes in both the cortex and hippocampus were altered in one COX isoform knockout mouse but not the other. The major gene function affected in all genotype comparisons was 'transcriptional regulation'. Distinct biologic and metabolic pathways that were altered in COX^-/- ^mice included β oxidation, methionine metabolism, janus kinase signaling, and GABAergic neurotransmission.

**Conclusion:**

Our findings suggest that COX-1 and COX-2 differentially modulate brain gene expression. Because certain anti-inflammatory and analgesic treatments are based on inhibition of COX activity, the specific alterations observed in this study further our understanding of the relationship of COX-1 and COX-2 with signaling pathways in brain and of the therapeutic and toxicologic consequences of COX inhibition.

## Abstract 2

Microarray analysis of gene expression in the cerebral cortex and hippocampus of mice deficient in cyclooxygenase-1 or cyclooxygenase-2 reveals that the two enzymes differentially modulate brain gene expression.

## Background

Prostaglandin H synthase, otherwise known as cyclooxygenase (COX), catalyzes the first metabolic step in the transformation of arachidonic acid (AA) to the bioactive products prostaglandins and thromboxanes [1]. The existence of two isoforms of prostaglandin H synthase, namely COX-1 and COX-2, has been confirmed in multiple organs, including brain [2,3]. Not only are these enzymes physiologically important in their role in AA metabolism but they are also important pharmacologic targets of analgesics and anti-inflammatory agents [3]. Mice deficient in either COX-1 (COX-1^-/-^) or COX-2 (COX-2^-/-^) are available and have been used to advance our understanding of the physiologic and pathologic roles of the individual COX isoforms [4].

Although it is known that in brain both COX-1 and COX-2 are expressed constitutively and that COX-2 can be induced upon the presence of an insult, complete understanding of the role of each individual isoform is lacking. Our laboratory has attempted to elucidate the role of each isoform on brain physiology by using COX-1^-/- ^and COX-2^-/- ^mice. We found that COX-2^-/- ^mice have altered expression and activity of enzymes in the AA metabolism cascade, including increases in COX-1, cytosolic phospholipase A_2 _(cPLA_2_) and secretory phospholipase A_2 _expression [5]. Similar alterations have been observed in COX-1^-/- ^mice, in which COX-2 protein expression and cPLA_2 _and secretory phospholipase A_2 _gene and protein expression are increased [6]. However, the levels of prostaglandin E_2_, which is one of the major end products of the COX reaction, were increased in COX-1^-/- ^mice but decreased in COX-2^-/- ^mice. In addition, it has also been shown that COX-1^-/- ^and COX-2^-/- ^mice exhibit profound differences in activation of the transcription factor nuclear factor-κB (NF-κB) [6,7]. Overall, these previous studies suggest that each isoform and their end products, which function through specific prostaglandin receptors, play a unique role in the regulation of gene expression in the brain.

It has also been shown that mice with genetic deletion of an individual COX isoform have altered responses to pathologic insults. For instance, COX-2^-/- ^mice are known to be more resistant to direct cortical injections of *N*-methyl-D-aspartate, middle cerebral artery occlusion (MCAO), and systemic injections of 1-methyl-4-phenyl-1,2,3,6-tetrahydropyridine (MPTP) [8,9]. However, the precise downstream molecular mechanisms involved in these processes are not clearly characterized. Therefore, it is quite clear that an understanding of the COX-1^-/- ^and COX-2^-/- ^mouse brain transcriptome is necessary to elucidate further the individual roles of COX-1 and COX-2 in both normal brain function and response to injury.

Although previously characterized alterations in gene and protein expression in COX-deficient mice have been examined, they were discovered in a 'one protein and one gene at a time fashion' using Western blotting and real-time polymerase chain reaction (PCR) [5-7]. The usage of high-throughput technology such as microarray analysis can enhance our ability to characterize the effect of deleting the expression of either COX-1 or COX-2 on the expression of networks of genes normally controlled by the end products of these individual COX isoforms. Therefore, we used microarray analysis with quantitative, real-time PCR (Q-PCR) validation to determine the effect of deletion of either COX-1 or COX-2 on the transcriptome of two separate regions of mouse brain, namely hippocampus and cerebral cortex. Further analysis of the entire dataset with Ingenuity Pathways analysis software (Ingenuity Systems, Redwood City, CA, USA), a web-based software application that assists in the analysis and elucidation of complex biologic systems, revealed specific networks of genes that were differentially regulated. We decided to focus on gene expression changes that comprised specific biologic functions, and not just individual genes that are affected by genetic deletion of individual COX isoforms. Our findings suggest that genetic ablation of COX activity alters the transcription of a multitude of genes. Furthermore, we demonstrate that genetic deletion of COX isoforms alters the expression of tandem genes that are involved in metabolic and signaling pathways such as β oxidation, methionine metabolism, γ-aminobutyric acid (GABA) neurotransmission, and cytokine signaling. We also describe genes differentially expressed in COX-1^-/- ^versus COX-2^-/- ^mice, suggesting a unique role for COX-1 or COX-2 in modulating gene expression.

## Results

### Differential gene expression in COX-1^-/- ^and COX-2^-/- ^mice

In the cerebral cortex, 94 genes were differentially expressed in COX-1^-/- ^mice and 157 in COX-2^-/- ^mice compared with respective wild-type mice. In the hippocampus, 182 genes were differentially expressed in COX-1^-/- ^mice and 168 in COX-2^-/- ^mice compared with wild-type mice. These genes represent the entire set of differentially expressed genes, including expressed sequence tags, and RIKEN and chromosomal segment sequences. Further description of this dataset includes only the subset of genes that were annotated with an ascribed function or gene name. A complete list of the annotated, differentially expressed genes for both brain regions and all genotype comparisons can be found in the Additional data files, and the entire raw dataset can be found in the Gene Expression Omnibus (GEO: GSE5342).

Using DRAGON (Database Referencing of Array Genes Online), SwissProt function labels were ascribed to each gene and genes were parsed into categories according to the function labels. In both COX-1^-/- ^and COX-2^-/- ^mice, the greatest number of genes whose expression was significantly changed in both brain regions belonged to the SwissProt function category of transcriptional regulation (cerebral cortex: COX-1^-/- ^= 12% and COX-2^-/- ^= 7%; hippocampus: COX-1^-/- ^= 11% and COX-2^-/- ^= 12%). COX-2^-/- ^mice exhibited significant changes in expression of genes belonging to the transmembrane (6%) and mitochondrion (5%) functional categories in cerebral cortex, whereas COX-1^-/- ^mice exhibited significant alteration in expression of genes classified as nuclear proteins (9%). In contrast, expression of genes belonging to the kinases (7%) functional group was selectively changed in the hippocampus of COX-1^-/- ^but not of COX-2^-/- ^mice.

Although a majority of the genes (>93%) shown to be differentially expressed in the separate brain regions of COX-1^-/- ^or COX-2^-/- ^mice only occurred in mice null for one isoform and not the other, the expression of some genes was changed in both COX-1^-/- ^and COX-2^-/- ^mice. Table 1 lists all of the genes whose expression was changed in both COX-1^-/- ^and COX-2^-/- ^mice, either in the same or in the opposite direction. In the cerebral cortex, there were four genes with altered expression in both COX-1^-/- ^and COX-2^-/- ^mice. Of these four, Rho-GDP dissociation inhibitor α exhibited increased expression in both genotypes. The expression of the other three genes (mitochondrial inner membrane protein, GABA transporter [GAT]3, and ring finger protein 24) changed in opposite directions (downregulated in COX-1^-/- ^and upregulated in COX-2^-/- ^mice), suggesting an isoform-specific effect on expression of these genes. Such an isoform-specific effect was not observed in the hippocampus, where all 12 genes whose expression was altered in both COX-1^-/- ^and COX-2^-/- ^mice exhibited changes in a similar direction in both genotypes when compared with wild-type mice (Table 1), suggesting that this cohort of genes is responsive to a general alteration in AA metabolism that is not specific to COX-1 or COX-2.

Upregulation of gene expression of cPLA_2_, which releases AA from phospholipids, as previously described by Q-PCR in whole brain of COX-1^-/- ^and COX-2^-/- ^mice [5,6], was not detected in cortex (COX-1: *z *ratio = 0.72, *P *= 0.03; COX-2: *z *ratio = 0.04, *P *= 0.9) and hippocampus (COX-1: *z *ratio = -0.89, *P *= 0.002; COX-2: *z *ratio = 0.60, *P *= 0.13) by microarray analysis. Although a thorough study regarding the expression of cPLA_2 _mRNA in mouse brain has not been undertaken, expression patterns of cPLA_2 _mRNA in rat brain suggest elevated expression in the midbrain, thalamus, hypothalamus, pons, and pineal gland compared with cerebral cortex and hippocampus [10]. Therefore, this heterogeneous expression of cPLA may account for the detection of differential regulation in whole brain but not in distinct regions, such as hippocampus and cortex.

**Figure 1:**
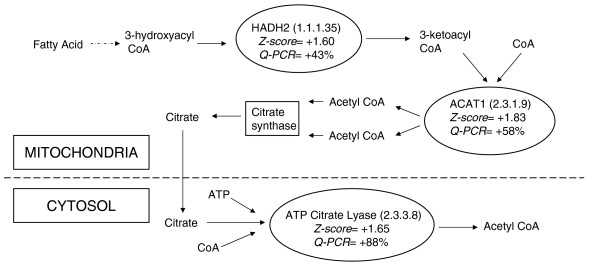
β Oxidation gene expression is increased in cerebral cortex of cyclo-oxygenase (COX)-2^-/- ^mice. The expression of three tandem genes involved in the metabolism of short chain fatty acids to citrate is increased in cerebral cortex of COX-2^-/- ^mice, as determined by microarray and quantitative PCR (Q-PCR) analysis. These genes, namely hydroxyacyl coenzyme-A dehydrogenase (HADH2), acetyl coenzyme-A acetyltransferase (ACAT1) and ATP citrate lyase, are depicted in the ovals above, along with the enzyme classification (EC) number in parenthesis. *Z *ratio from the microarray analysis and Q-PCR validation results for each gene are provided below the name of the gene. A positive *z *ratio represents an increase in expression in COX knockout mice, whereas a negative value denotes a decrease in expression in COX knockout mice. Q-PCR percentage represents the percentage increase in expression over wild-type mice. HADH2 and ACAT1 are expressed in the mitochondria and ATP citrate lyase is expressed in the cytosol. The expression of genes represented as a square is not changed.

**Figure 2:**
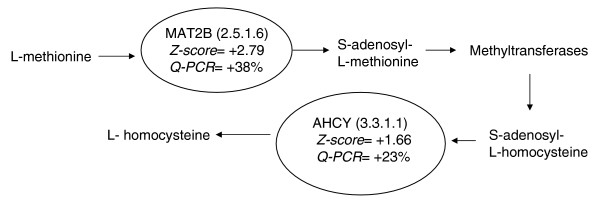
Methionine metabolism gene expression is increased in the cerebral cortex of cyclo-oxygenase (COX)-2^-/- ^mice. The expression of two tandem genes involved in the metabolism of methionine to homocysteine is increased in the cortex of COX-2^-/- ^mice, as determined by microarray and quantitative PCR (Q-PCR) analysis. These genes, namely methionine adenosyltransferase (MAT2B) and S-adenosylhomocysteine hydrolase (AHCY), are depicted in the ovals above, along with the enzyme classification (EC) number in parenthesis. *Z *ratio from the microarray analysis and Q-PCR validation results for each gene are provided below the name of the gene. A positive *z *ratio represents an increase in expression in COX knockout mice, whereas a negative value denotes a decrease in expression in COX knockout mice. Q-PCR percentage represents the percentage increase in expression over wild-type mice.

Using Ingenuity Pathways analysis software, we identified specific networks of genes that were differentially regulated. These networks of genes constituted specific metabolic and signaling pathways, including β oxidation of lipids (Figure 1), methionine metabolism (Figure 2), GABAergic neurotransmitter signaling (Table 2), and a COX isoform specific effect on Janus kinase (JAK) expression (Table 2).

### Expression of genes involved in beta oxidation is upregulated in cortex of COX-2^-/- ^mice

In cerebral cortex of COX-2^-/- ^mice, we identified increased expression of three genes that work in tandem in the final steps of short chain lipid metabolism by β oxidation, namely hydroxyacyl-coenzyme A dehydrogenase type II (HADH2), acetyl-coenzyme A acetyltransferase 1 (ACAT1) and ATP citrate lyase (ACLY; Figure 1). Expression of HADH2 was determined to be increased in microarray analysis of cerebral cortex of COX-2^-/- ^mice with a *z *ratio of +1.60. Q-PCR also demonstrated an increase in HADH2 expression (143% ± 24% in COX-2^-/- ^mice versus 100% ± 20% in wild-type mice; *t *statistic = -3.26, *P *= 0.01). ACAT1 expression was also increased in cerebral cortex of COX-2^-/- ^mice (*z *ratio = +1.83), which was validated by Q-PCR analysis (158% ± 19% in COX-2^-/- ^mice versus 100% ± 10% in wild-type mice; *t *statistic = -6.13, *P *= 0.0003). Finally, ACLY expression was increased in microarray analysis of the cerebral cortex of COX-2^-/- ^mice (*z *ratio = +1.65), and the increase was also validated with Q-PCR (188% ± 33% in COX-2^-/- ^mice versus 100% ± 49% in wild-type mice; *t *statistic = -3.41, *P *= 0.008). Gene expression of ACAT 1, but not that of HADH2 and ACLY, was found to be decreased in the hippocampus of COX-1^-/- ^mice (z ratio= -3.00; 72% ± 6% in COX-1^-/- ^mice versus 100% ± 21% in wild-type mice; *t *statistic = 2.99, *P *= 0.03).

### Expression of genes involved in methionine metabolism is upregulated in cortex of COX-2^-/- ^mice

Expression of methionine adenosyltransferase II (MAT2B) and adenosylhomocysteine hydrolase (AHCY), two genes that work in tandem to metabolize methionine to homocysteine, was increased in cerebral cortex of COX-2^-/- ^mice (Figure 2). MAT2B expression was found to be increased in microarray analysis (*z *ratio = +2.79) and by Q-PCR analysis (138% ± 12% in COX-2^-/- ^mice versus 100% ± 25% in wild-type mice; *t *statistic = -3.14, *P *= 0.013). Increased AHCY expression, as detected by microarray analysis (*z *ratio= +1.66), was validated by Q-PCR (123% ± 5% in COX-2^-/- ^mice versus 100% ± 14% in wild-type mice; *t *statistic = -3.76, *P *= 0.01). Expression of MAT2B, but not of AHCY, was decreased in COX-1^-/- ^hippocampus (*z *ratio = -3.07; 78% ± 13% in COX-1^-/- ^mice versus 100% ± 18% in wild-type mice; *t *statistic = 2.39, *P *= 0.04).

### GABA transporter and receptor expression is altered in COX^-/- ^mice

Expressions of two genes that are involved in GABA neurotransmission were altered in cerebral cortex and hippocampus of COX knockout mice. Microarray analysis of GABA-A receptor subunit β_1 _(GABRB1) in the hippocampus of COX-2^-/- ^mice demonstrated downregulation (*z *ratio = -2.32) of gene expression (Table 2). Validation with Q-PCR demonstrated that the expression of GABRB1 was not only decreased in hippocampus of COX-2^-/- ^mice (54% ± 18% in COX-2^-/- ^mice versus 100% ± 19% in wild-type mice; *t *statistic = 4.08, *P *= 0.003) but it was also decreased in cerebral cortex of COX-2^-/- ^mice, a change that was not initially detected by microarray analysis (72% ± 8% in COX-2^-/- ^mice versus 100% ± 17% in wild-type mice; *t *statistic = 3.67, *P *= 0.008).

GABA transporter (GAT)3 expression in cerebral cortex of COX^-/- ^mice exhibited a genotype-specific effect (Table 2). COX-1^-/- ^mice demonstrated downregulation of gene expression (*z *ratio = -1.86) whereas COX-2^-/- ^showed upregulation of mRNA level (*z *ratio = +1.68). Validation with Q-PCR demonstrated the same pattern of expression in COX-1^-/- ^mice (58% ± 12% in COX-1^-/- ^mice versus 100% ± 22% in wild-type mice; *t *statistic = 3.70, *P *= 0.006) and COX-2^-/- ^mice (166% ± 37% in COX-2^-/- ^mice versus 100% ± 13% in wild-type mice; *t *statistic = -3.82, *P *= 0.01). No significant difference in the expression of GAT3 was detected by microarray or Q-PCR in the hippocampus of any mice examined in the present study (data not shown).

### Janus kinase expression is altered in a genotype-dependent manner in hippocampus

Janus kinase (JAK) isoforms 1 and 2 were found to be expressed in a genotype-dependent manner in hippocampus (Table 2). COX-1^-/- ^mice had increased expression of JAK1 (*z *ratio = +1.56) but not of JAK2. Q-PCR validation also demonstrated increased expression of JAK1 in COX-1^-/- ^mice (196% ± 79% in COX-1^-/- ^mice versus 100% ± 40% in wild-type mice; *t *statistic = -2.45, *P *= 0.04). On the other hand, JAK-2 expression was decreased in COX-2^-/- ^but not in COX-1^-/- ^mice (*z *ratio = -2.09). Validation with Q-PCR confirmed this selective decrease (82% ± 12% in COX-2^-/- ^mice versus 100% ± 10% in wild-type mice; *t *statistic = 2.54, *P *= 0.03). No significant differences in expression of JAK-1 or JAK-2 were observed in cerebral cortex (data not shown).

## Discussion

In this study, we demonstrate that COX-1^-/- ^or COX-2^-/- ^mice exhibit significant changes in the hippocampus and cerebral cortex transcriptome. Some differentially expressed genes occur in both genotypes but most changes observed (>93%) are unique to one isoform deletion and not the other. This observation implies that despite the functional similarities of the two COX isoforms in the metabolism of AA to eicosanoids, they impact the basal physiology of the brain in different ways. Because the end products of COX-mediated AA metabolism, through the subsequent action of thromboxane and prostaglandin synthases, are the bioactive mediators of COX activity, this study suggests that COX-1 or COX-2 specific end products differentially affect the mouse brain transcriptome.

### Altered expression of mitochondrial function and β oxidation genes

The selective changes in transmembrane and mitochondrial genes in COX-2^-/- ^but not COX-1^-/- ^may be related to the different subcellular localization of the two COX isoforms [11]. The deletion of COX-2, normally localized at nuclear envelope [11], might affect the expression of genes directly or indirectly coupled with COX-2. Although coupling between COX-2 and mitochondrial enzymes in brain has not been reported, mitochondrial localization of COX-2 has been reported in cancer cells and in corpus luteum [12,13]. Our observation that important genes involved in mitochondrial energy metabolism are upregulated in the cerebral cortex of COX-2^-/- ^mice suggests that COX-2 is critical for mitochondrial function.

Our findings demonstrate that expressions of HADH2, ACAT1, and ACLY mRNA were upregulated in the cerebral cortex of COX-2^-/- ^mice. The protein products of these genes work in tandem to metabolize lipids, through the process of β oxidation, to acetyl-coenzyme A [14-16].

The consequences of an increase in brain expression of three major enzymes involved in the β oxidation of lipids implies an increase in β oxidation of fatty acids in COX-2^-/- ^mice. Supporting this hypothesis, a previous study examining the incorporation of AA in the brains of COX-2^-/- ^mice [17] suggested that the increase in baseline incorporation of AA in awake COX-2^-/- ^mice is possibly due to increased β oxidation. Because a component of the kinetic model used by Rapoport and coworkers allows for the possibility of a portion of the infused AA to be shunted to β oxidation [18], it is possible that the increased incorporation of AA in the brains of COX-2^-/- ^mice represents increased β oxidation mediated by an increase in HADH2, ACAT1, and ACLY. However, it remains unclear why the deletion of COX-2, but not COX-1, would result in an increase in β oxidation enzymes in the brain.

Although these enzymes function in tandem in the β oxidation pathway of lipid metabolism, HADH2 has been shown to play a role in modifying the susceptibility of mouse brain to certain insults. Transgenic mice overexpressing HADH2 have proved to be more resistant to the brain damage associated with MCAO and MPTP [8,9]. Similarly, COX-2^-/- ^mice also exhibit increased resistance to MPTP and MCAO induced neuronal damage [8,9]. It is unclear whether increased expression of HADH2 in COX-2^-/- ^mice, as observed in the present study, contributed to those previous findings, but it is interesting that mice overexpressing HADH2 and COX-2^-/- ^mice are both resistant to the aforementioned nervous system insults.

### Altered expression of homocysteine metabolism genes in the cerebral cortex of COX-2^-/- ^mice

Our study demonstrated that the expressions of MAT2B and AHCY mRNA, the protein products of which are two tandem enzymes involved in methionine metabolism, are upregulated in the cerebral cortex of COX-2^-/- ^but not of COX-1^-/- ^mice.

MAT2B increases the level of product inhibition of the MAT holoenzyme [19,20]. Because increased levels of *S*-adenosylmethionine are observed when MAT2B is downregulated [19], we could predict that increased expression of MAT2B, as observed in our study, would result in decreased S-adenosylmethionine, which is the exclusive methyl donor for transmethylation reactions [19-21].

Being the sole enzyme known to metabolize *S*-adenosylhomocysteine, which is the product of transmethylation reactions, AHCY regulates all *S*-adenosylmethionine dependent methyltransferases [22]. Increased expression of AHCY in the cortex of COX-2^-/- ^mice is predicted to increase the production of adenosine and homocysteine [23]. The suggestion that an increase in homocysteine production may occur in COX-2^-/- ^mice is intriguing because homocysteine is known to activate endogenous glutamate receptors and potentiate the effect of certain excitotoxins such as kainic acid [24,25]. The possibility that increased expression of AHCY results in elevated homocysteine concentrations is consistent with a preliminary observation made in our laboratory indicating that COX-2^-/- ^mice, but not COX-1^-/- ^mice, have increased seizure intensity and neuronal damage after systemic injection with kainic acid [26]. Our findings support the observation that homocysteine pretreatment increases the intensity of kainate-induced seizures and neuronal damage [25].

### Altered expression of GABA neurotransmission genes in COX-1^-/- ^and COX-2^-/- ^mice

We have identified two differentially expressed genes, GABRB1 and GAT3, from the cortical and hippocampal GABAergic system of COX deficient mice. GABRB1 is a β subunit of the GABA_A _ionotropic receptor ion channel [27]. GABRB1 mRNA is known to be downregulated by persistent GABA_A _receptor activation and in a model of temporal lobe epilepsy [28,29]. It is well established that a decrease in GABA receptor number is preceded by a decrease in GABA receptor subunit mRNA [30]. Therefore, our finding that GABRB1 mRNA expression is decreased suggests a downregulation of GABA ionotropic receptor in the hippocampus and cerebral cortex of COX-2^-/- ^mice but not COX-1^-/- ^mice.

Coupled with changes in GABRB1 in COX-2^-/- ^mice, we also demonstrated an alteration in the expression of a GABA transporter. This transporter, GAT3, is thought to terminate the GABA signal by transporting GABA from the synapse into the cell [31-33]. The alteration in GAT3 expression observed in the present study suggests an increased level of inhibitory input in COX-1^-/- ^mice, whereas COX-2^-/- ^mice may have less inhibitory input because of altered rates of GABA uptake from the synapse. In fact, increased expression of GAT3 has been demonstrated to be associated with epileptogenic hippocampal tissue [34]. Furthermore, it has been shown that overexpression of GAT in transgenic mice increases their susceptibility to kainate-induced seizures [35].

The combined effect of decreased expression in GABRB1 and an increase in GAT3 in COX-2^-/- ^mice, but not COX-1^-/- ^mice, is also consistent with preliminary data from our group demonstrating an increased susceptibility of COX-2^-/- ^mice, but not COX-1^-/- ^mice, to kainate induced excitotoxicity [26]. The net effect of the alterations in GABA-related gene expression in the COX-2^-/- ^mouse brain would suggest decreased GABAergic tone, predisposing these mice to an increased neuronal excitability that would increase their susceptibility to excitotoxins such as kainate. Although our observations in the present study are consistent with our previous data [26], it remains to be determined how deletion of COX-2, but not of COX-1, alters the expression of GABAergic system related genes.

### COX genotype-dependent alteration of Janus kinase isoforms in the mouse hippocampus

JAK1 and JAK2 are non-receptor tyrosine kinases that are essential for the signal transduction of cytokine receptor activation (For reviews, see Aringer and coworkers [36] and Leonard and O'Shea [37]). Both isoforms are ubiquitously expressed but are involved in the transduction of specific groups of cytokine signals [36,37]. Furthermore, JAK1 and JAK2 knockout mice demonstrate the importance and nonredundancy of these JAK isoforms, because both mutations are lethal [36].

We found that JAK1 mRNA expression, but not that of JAK2, is increased in the hippocampus of COX-1^-/- ^mice, and that JAK2, but not JAK1, is decreased in the hippocampus of COX-2^-/- ^mice. Although this is the first report to demonstrate a JAK alteration in COX^-/- ^mice, COX-2 inhibitors have been shown to dampen interleukin-12 signaling by inhibiting the activation of JAK2 [38]. Although it is unclear how these alterations affect the brain physiology of the knockout mice, one could predict a different response of COX-1^-/- ^and COX-2^-/- ^mice to neuroinflammation caused by the role played by JAK in regulating inflammation-induced signaling. Interestingly, the pattern of change in JAK expression is similar to what has been observed for NF-κB in the brains of COX-1^-/- ^and COX-2^-/- ^mice [6,7]. COX-1^-/- ^mice exhibit an upregulation of the activity and expression of NF-κB accompanied by an increase in I-κB phosphorylation [6]. Conversely, COX-2^-/- ^mice exhibit decreased expression and activation of NF-κB accompanied by a decrease in I-κB phosphorylation [7]. Although the mechanism of crosstalk between the JAKs and NF-κB has not been elucidated, evidence suggests that JAK may be required for NF-κB activation [39]. In this regard, the similarity in direction of expression of JAK and NF-κB based on COX isoform deficiency is intriguing and requires further study.

## Conclusion

The present study is the first to demonstrate the specific effect of genetic ablation of COX-1 or COX-2 on the mouse brain transcriptome. Our findings suggest that ablation of COX activity alters the transcription of many genes, including those involved in β oxidation, methionine metabolism, GABA neurotransmission, and cytokine signaling. Although some of the molecular mechanisms underlying these changes are not well understood at this time, these data identify metabolic and signaling pathways that were previously not known to be affected by COX. Because many anti-inflammatory and analgesic treatments, such as nonsteroidal anti-inflammatory drugs, rely on reduction in COX activity for their mechanism of action, the specific alterations observed in this study expand our understanding of the therapeutic and toxicologic consequences of COX inhibition.

## Materials and methods

### Animal procedure

COX-1^-/- ^or COX-2^-/- ^mice and respective wild-type mice (C57BL6/6-129/Ola mixed genetic background) [4] were received at the animal facility of the National Institute on Aging at 6 weeks of age from the National Institute of Environmental Health Sciences colony maintained by Taconic Farms (Germantown, NY, USA), with heterozygous by heterozygous breedings for more than 35 generations. The heterozygotes are maintained independently for each COX line. F_1 _offspring of these heterozygous × heterozygous matings are then mated wild-type × wild-type to generate the wild-type mice used in the study. Male homozygous null mice (-/-) are mated with heterozygous female mice (+/-) to generate the -/- mice used in the present study. Thus, all mice used in the study were one generation from the heterozygous × heterozygous maintenance colony. COX-1^-/- ^and COX-2^-/- ^mice were then compared with respective wild-type mice (COX-1^+/+ ^and COX-2^+/+^, respectively) for microarray and Q-PCR analysis.

Mice were housed at room temperature on a 12 hour light/dark cycle with free access to food and water in a ventilated cage rack system. At 12 weeks of age the mice were killed with an overdose of sodium pentobarbital (100 mg/kg) followed by decapitation. Brains were quickly removed, dissected, and stored at -80°C until usage. All procedures involving mice were approved by the National Institutes of Child Health and Human Development Animal Care and Use Committee, and were performed in accordance with National Institutes of Health guidelines on the care and usage of laboratory animals.

### RNA extraction and cDNA synthesis

Fresh frozen mouse hippocampus and cerebral cortex were processed for RNA extraction using the Qiagen RNeasy Lipid Tissue Mini kit (Qiagen, Valencia, CA, USA) under RNAse-free conditions by following the manufacturer's suggested procedure. RNA purity and integrity were verified by examining the 260 nm/280 nm ratio using a spectrophotometer and an ethidium bromide-agarose gel imaged under UV light, respectively. Extracted RNA was resuspended in RNAse-free molecular grade water and stored at -80°C until use. For Q-PCR, total RNA (5 μg) was reverse transcribed using a High Capacity cDNA Archive kit (Applied Biosystems, Foster City, CA, USA) using appropriate controls to ensure the absence of genomic DNA contamination.

### Microarray procedures

Microarray procedure protocols used in the study are described in the National Institute on Aging Gene Expression and Genomic Unit website [40]. Briefly, 5 μg total RNA was reverse transcribed in the presence of ^33^P-dCTP, and labeled cDNA was purified using a Biospin P-30 column (Bio-Rad, Hercules, CA, USA). A custom 17 K mouse nylon membrane microarray (GEO: GPL4006) was used for the high throughput analysis, which was performed in triplicate for each sample. Labeled cDNA from cerebral cortex and hippocampus of COX-1^-/-^, COX-2^-/-^, COX-1^+/+^, and COX-2^+/+ ^(three mice per group) was hybridized to the nylon array for 16 to 18 hours. Arrays were washed, dried, and apposed to a phosphoimager screen for collection of data. Differentially expressed genes were identified by comparing the microarray data from COX-1^+/+ ^with those from COX-1^-/- ^mice and, separately, by comparing the data from COX-2^+/+ ^with those from COX-2^-/- ^mice (as described below). Microarray images were processed using ArrayPro (MediaCybernetics, Silver Spring, MD, USA); the raw data were transferred to an Excel spreadsheet and normalized using an Excel macro to convert raw data to *Z *normalization, as previously described, with the following equation [41]:

*Z *(raw data) = (ln [raw data] - avg [ln(raw data)])/(std dev [ln(raw data)])

Where avg is the average of all genes of an array, and std dev is the standard deviation of all genes of an array. This conversion compresses the dataset onto a log scale, normalizes the data to be symmetric around zero, and results in a standard deviation of 1, allowing data to be compared between arrays. Differentially expressed genes were identified using DIANE 1.0, a JMP (SAS Institute, Inc., Cary, CA, USA) based program developed by VVP. (Information on availability and components of DIANE can be found on the web [42]). DIANE 1.0 takes three factors into account to identify differentially expressed genes. First, for a gene to be considered differentially expressed, it must have similar values (both direction of change and magnitude) between replicate arrays, as assessed by the *Z *test, which results in a *P *value that is calculated using a normal distribution [41]. Second, the fold change between treatments is determined by *z *ratio:

*z *ratio (between treatment A and B) = (z [a] - z [b])/std dev

Where std dev is the standard deviation of all genes on an array. Because the *z *normalized data have a standard deviation of exactly 1, a significant fold change was considered to be represented by any change greater than 1.5 times the standard deviation, that is, a *z *ratio greater than 1.5. This threshold has been stated to result in consistent detection of differentially expressed genes [41]. To avoid detection of background level genes that may produce high fold changes and therefore contribute to false detection rates, we eliminate all genes below background by ensuring that the average intensity of a gene over replicates of treatment and control exceeds zero. This is enabled by the fact that the mean of *z *normalized arrays is always zero. Once processed as above, the data can be divided into five different significance levels: significance level +2 = *z *ratio >1.5 and significant replication (*P *< 0.05); significance level +1 = *z *ratio >1.5 and no significant replication (*P *> 0.05); significance level 0 = neither condition is true; significance level -1 = same as +1 but wild type > knockout; and, finally, significance level -2 = same as +2 but wild type > knockout

Genes that attain significance levels of +2 and -2 are highly significant and are considered differentially expressed between experimental groups. These genes are imported to Excel from DIANE according to name, symbol, gene accession number, function, significance level, and *z *score. Further annotation and analysis of the microarray dataset was performed using DRAGON [43] and Ingenuity Pathways (Ingenuity Systems, Redwood City, CA, USA). We did not explicitly calculate false-positive or false-negative rates for our microarray analysis because we used the *z *normalization primarily as a filter for selecting candidate genes for Q-PCR validation, which was carried out as described below.

### Quantitative PCR analysis

A total of nine genes detected by microarray analysis were validated using Q-PCR. These genes were selected because they were differentially expressed in the microarray analysis. In addition, these genes were selected for further validation because they comprised distinct biologic functions or signaling pathways, as determined by analysis using the Ingenuity Pathways software.

Validation of microarray results (*n *= 5-6/group) was performed using Q-PCR, with the ABI PRISM 7000 Sequence Detection System (Applied Biosystems). To increase the sample size and further validate our microarray results, to the same set of RNA samples used for microarray analysis (*n *= 3), a new set of RNA samples obtained from independent animals (*n *= 3) was added for each experimental group for Q-PCR analysis. In order to validate both positive and negative findings of the microarray analysis completely, all possible comparisons for genotype were performed in each brain region for each gene described below. All changes detected by microarray analysis were validated by Q-PCR. However, only changes in gene expression detected by Q-PCR that were statistically significant are described in the results section, whereas Q-PCR analyses that did not detect significant differential gene expression are provided in Additional data files.

Assay-on-Demand (Applied Biosystems) primers for ACAT1 (Unigene ID-Mm.293233), MAT2B (Unigene ID-Mm.293771), AHCY (Unigene ID-Mm.330692), HADH2 (Unigene ID-Mm.6994), GAT3 (Unigene ID-Mm.258596; also known as solute carrier family 6, member 13), GABRB1 (Unigene ID-Mm.226704), JAK1 (Unigene ID-Mm.289657), JAK2 (Unigene ID-Mm.275839), and ACLY (Unigene ID-Mm.282039) were used for Q-PCR validation of microarray results. Q-PCR results for the above listed genes were normalized to phosphoglycerate kinase 1 expression levels, as previously reported [5,6]. Briefly, Taqman Universal PCR Master Mix, Assay-On-Demand primers, and cDNA samples were mixed in RNAse-free water and added to an optical 96-well reaction plate (Applied Biosystems). Negative controls containing no cDNA and a standard curve spanning three orders of magnitude of dilution were run on each plate in duplicate. Q-PCR conditions were 50°C for 2 min and 95°C for 10 min, followed by 40 cycles of 15 s at 95°C and 1 min at 60°C. The amount of target gene expression was calculated by using either the ΔΔC_T _method [44] or by using the standard curve to determine the absolute quantity of transcript. Following the method of previous reports [5,6], relative expression values of genes are expressed as percentage of expression in wild-type mice.

### Statistical analysis

Data are expressed as *z *ratio of microarray data or mean percentage difference ± standard deviation from gene expression values in wild-type mice. Statistical analysis for Q-PCR data was performed using the freeware program Open Stat 4 [45]. For all comparisons, the F test for homogeneity of variance was first calculated. Depending on the results of the F test, an unpaired *t*-test assuming equal variance or an unpaired *t*-test assuming unequal variance was calculated. The *t *statistic and *P *value for each comparison is reported. *P *< 0.05 was considered statistically significant.

## Additional data files

The following additional data are available with the online version of this article. Additional data file 1 provides a full list of differentially expressed genes in cerebral cortex. Additional data file 2 provides a full list of differentially expressed genes in hippocampus. Additional data file 3 provides raw data for all Q-PCR analyses.

## Supplementary Material

Additional data file 1 A full list of differentially expressed genes in cerebral cortex. Click here for file

Additional data file 2 A full list of differentially expressed genes in hippocampus. Click here for file

Additional data file 3 Raw data for all Q-PCR analyses. Click here for file

## Figures and Tables

**Table 1:** Genes whose expression is altered in both COX-1^-/- ^and COX-2^-/- ^mice

Genbank accession number	Gene name	COX-1^-/- ^versus wild type	COX-2^-/- ^versus wild type
Cortex			
BG086892	Rho GDP dissociation inhibitor (GDI) α	2.40	1.69
AW555640	Inner membrane protein, mitochondrial	-5.49	2.16
BG075861	Solute carrier fam. 6 (neurotransmitter transporter, GABA), mem. 13	-1.86	1.68
BG085367	Ring finger protein 24	-2.84	2.15
Hippocampus			
BG064367	Ectonucleoside triphosphate diphosphohydrolase 1	-1.84	-1.94
BG064631	Timeless homolog (*Drosophila*)	1.95	1.81
BG065181	RNA binding motif, single stranded interacting protein 1	1.52	2.09
BG065688	Ubiquitin A-52 residue ribosomal protein fusion product 1	3.21	2.62
BG066667	Microtubule-associated protein 4	1.6	2.07
BG067592	Nuclear receptor corepressor 2	2.13	1.54
BG067766	Nuclear receptor subfamily 1, group H, member 2	1.65	1.51
BG069493	Rho-guanine nucleotide exchange factor	-1.84	-1.52
BG078482	Synaptotagmin binding, cytoplasmic RNA interacting protein	1.67	1.84
BG079093	Glycolipid transfer protein	2.26	2.02
BG081977	THAP domain containing 11	-1.85	-1.94
BG083840	Mitogen-activated protein kinase 13	2.06	2.23

Gene names are provided with GenBank accession numbers and *z *ratio. A positive z-ratio represents an increase in expression in COX^-/- ^mice while a negative value denotes a decrease in expression in COX^-/- ^mice. COX, cyclooxygenase.

**Table 2:** Expression of GABA and JAK signaling-related genes in brain of COX knockout mice

Gene name	Region	COX-1^-/-^	COX-2^-/-^
GAT3	Cerebral cortex	-42% (-1.86)	+66% (+1.68)
GABRB1	Cerebral cortex	-	-28% (ND)
GABRB1	Hippocampus	-	-46% (-2.32)
JAK 1	Hippocampus	+96% (+1.56)	-
JAK 2	Hippocampus	-	-18% (-2.09)

Percentage change in expression, as validated by Q-PCR, is provided, along with microarray-determined *z *ratios (in parenthesis) for each genotype comparison. Janus kinase (JAK)1 and 2 expression are altered only in hippocampus of COX-1^-/- ^and COX-2^-/- ^mice. COX, cyclooxygenase; GABA, γ-aminobutyric acid; GABRB1, GABA-A receptor subunit β_1_; GAT, GABA transporter; ND, not differentially expressed in microarray analysis.
